# Tibia Tuberosity Fracture and Patellar Tendon Rupture Combination in a Pediatric Patient: Case Report and Narrative Review

**DOI:** 10.7759/cureus.24182

**Published:** 2022-04-16

**Authors:** Vasileios K Mousafeiris, Thomas Repantis, Nektaria Kalyva, Ioannis Papaioannou, Christine Arachoviti, Stamatia Chatziperi, Andreas Baikousis

**Affiliations:** 1 Orthopedics and Traumatology, General Hospital of Patras "Agios Andreas", Patras, GRC; 2 Pediatrics, University Hospital of Patras, Patras, GRC

**Keywords:** sports injury, pediatric trauma, patellar tendon rupture, tibia tubercle fracture, tibia tubercle avulsion, patellar tendon, tibia tubercle

## Abstract

A combination of tibial tuberosity (TT) fracture (TTF) along with patellar tendon (PT) rupture (PTR) is rare. We report a 15-year-old male who presented to our ED with acute knee pain and an inability to actively extend the knee after jumping during a basketball game. Diagnosis of simultaneous PTR is crucial as it changes clinical management. It is, therefore, important to maintain a high index of suspicion for the combination of TTF and PT injury.

## Introduction

Sports injuries in children are common and are getting diagnosed more frequently as more children have access to team sports, and the diagnostic tools are more precise. The international medical literature also helps in this direction, as more physicians are becoming aware of various types of sports injuries and how to manage them.

Tibial tuberosity fracture, in particular, has been described in the literature as secondary to an injury of the physeal growth plate [1-3]. It is seen more frequently in boys and associated with sports such as basketball and football that include excessive jumping activity [3]. It has been described either after forceful contraction of the quadriceps during jumping or eccentric quadriceps contraction during forceful knee flexion [2].

Although isolated tibial tuberosity (TT) fracture (TTF) or patellar tendon (PT) rupture (PTR) have been described, the combination of TTF along with PTR is rare. We hereby present a rare case of TTF with simultaneous PTR after jumping action during basketball training. The purpose of the report is not only to discuss the related literature but, most importantly, to raise the index of suspicion among all orthopedic surgeons in order to avoid underdiagnosis and undertreatment of these complex injuries.

## Case presentation

A 15-year-old male presented to the trauma emergency department of our hospital due to an injury to his left knee while playing sports. His injury occurred while he was playing basketball, and he attempted for lay-up. Subsequently, the patient experienced severe pain in the left knee, fell directly onto the ground, and could not bear weight on his left leg.

The patient's medical history was unremarkable, apart from a fracture of the left femur after a fall at the age of 18 months, which was treated conservatively. No previous complaints of pain or any other problem regarding his left knee were reported. His BMI (19 kg/m^2^) was normal.

During a physical examination, the skin on the left knee was intact, without abrasions or wounds, and the patient was unable to actively extend the knee. Swelling around the knee, moderate joint effusion, and severe pain during palpation of the TT (8/10 on the visual analogue scale) were noticed. The injured patella was also asymmetrically elevated compared to the contralateral patella. The Lachman test result was negative, and there was no knee instability. The affected extremity was neurovascularly intact.

**Figure 1:**
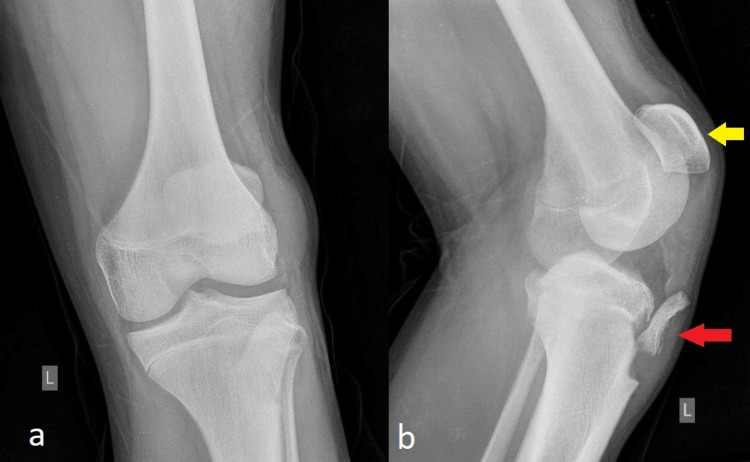
Anteroposterior (a) and lateral (b) radiographs of the injured knee Avulsion fracture of the tibia tubercle (red arrow) and high-riding patella (patella alta; yellow arrow) are noted.

Plain anteroposterior (AP) and lateral radiographs revealed a displaced avulsion TTF extending to the level of the proximal tibial physis (Ogden type II) and a concomitant high-riding patella (patella alta; see Figure 1). 

Given the latter finding, concern was raised for PT type IIC injury.

The patient was treated operatively on the following day. Open reduction and internal fixation of the fracture and repair of the tendon injury were performed under general anesthesia.

The patient was positioned supine on a radiolucent table. After sterile preparation and draping with the knee in 30º of flexion, an anterior longitudinal incision extending from the distal pole of the patella to 2 cm below the TT was performed. Dissection was performed through the subcutaneous tissue to the extensor mechanism, where the fracture and hematoma were encountered.

**Figure 2:**
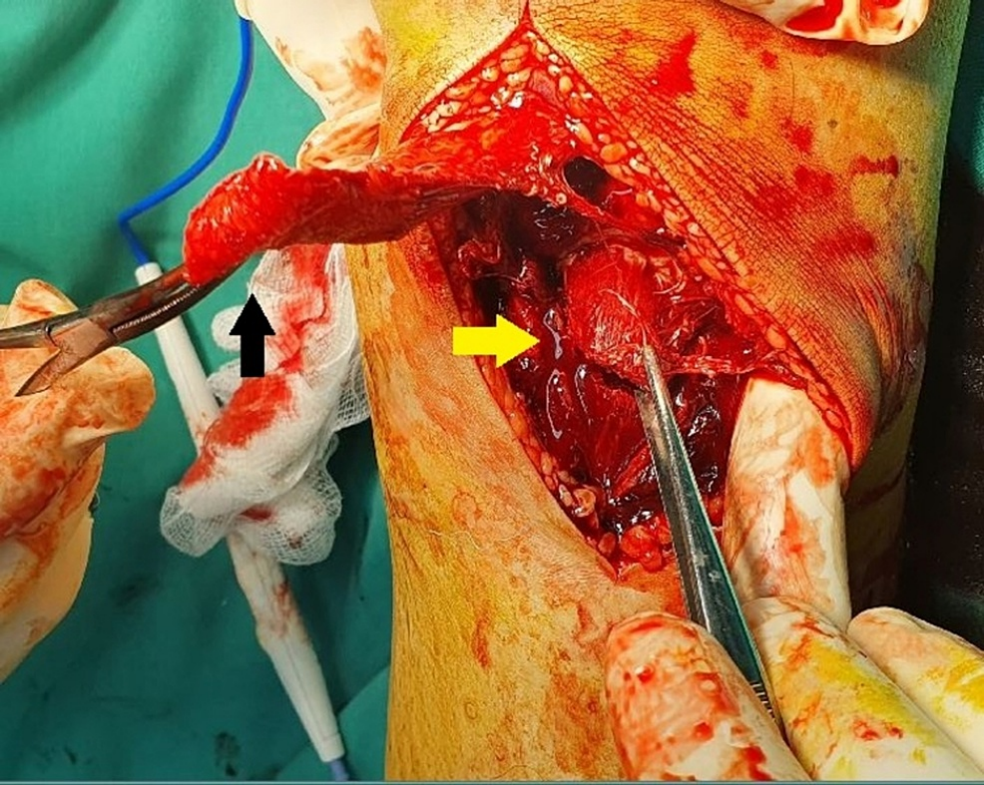
Intraoperative picture showing the rupture of the patellar tendon (black arrow) and the fracture line of the tibial tubercle (yellow arrow)

The PT was located for later reconstruction. Then the TT was found to be a completely free fragment, rotated by 180º and displaced from its original position (Figure 2).

**Figure 3:**
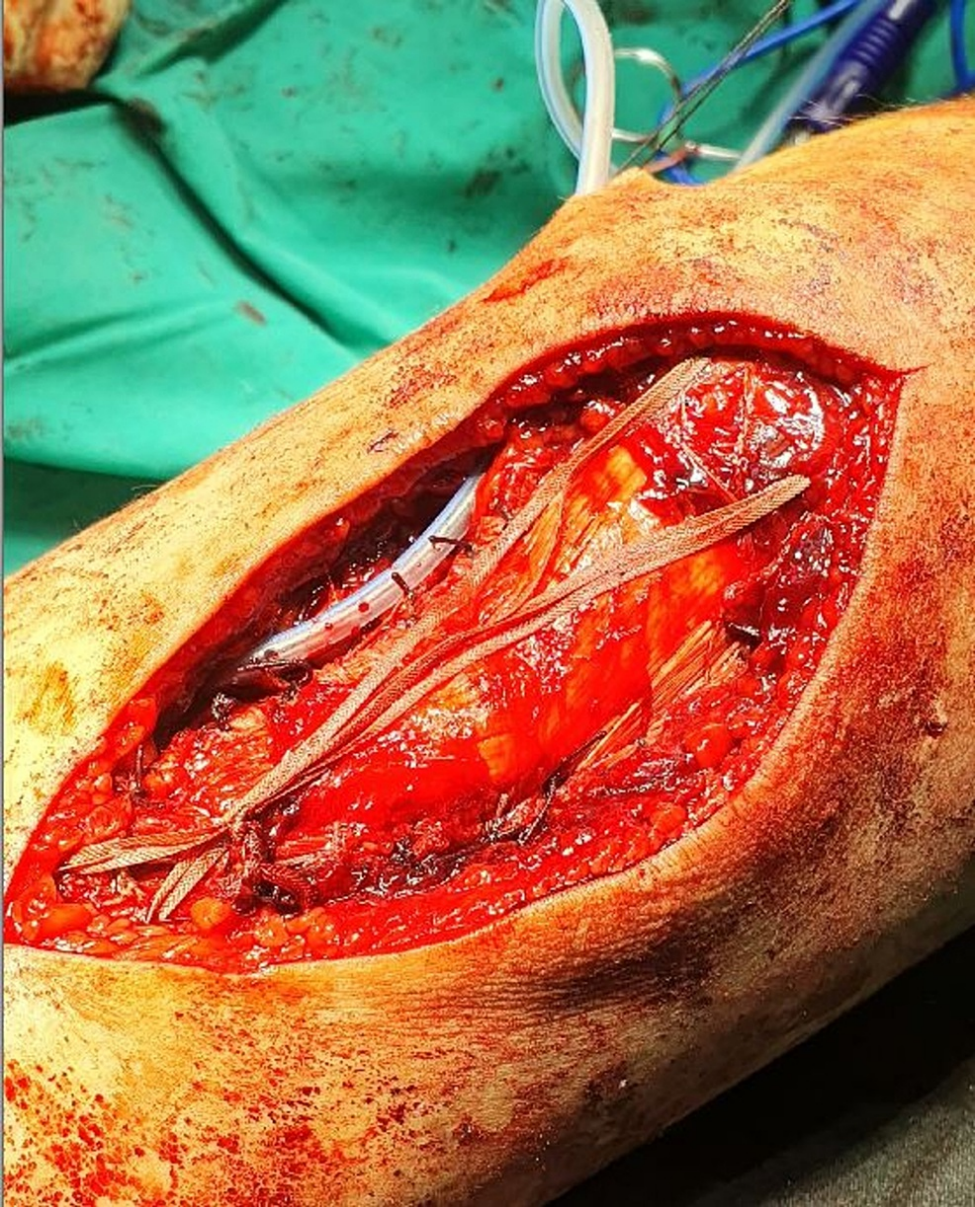
Intraoperative picture showing the placement of the patellar tendon back in its position

**Figure 4:**
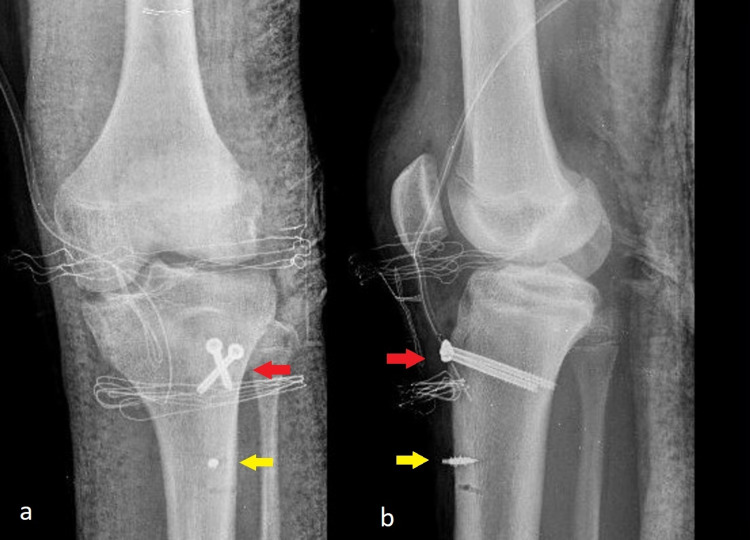
Immediate postoperative AP (a) and lateral (b) radiographs The two cannulated screws (red arrows) and the 5 mm anchor below the tibial tubercle (yellow arrows) are noted. AP - anteroposterior

The free TT edges were cleared of soft tissue, and the hematoma was removed. Then the PT was identified and completely avulsed from its distal native footprint. Macroscopically, the PT appeared delaminated and frayed without any intratendinous ruptures. Under fluoroscopic assistance, the TT was correctly reduced. The reduction was secured with a 0.045-inch K-wire and two half-threaded 6.5 mm × 50 mm cannulated screws (IRENE®, TianJin ZhengTian Medical Instrument Co., Ltd., China) with washers. Subsequently, PT reconstruction was performed. With the knee in 30º of flexion, the PT was reduced to its original footprint over the TT. The periosteum attached to the undersurface of the tendon was reapproximated onto its footprint on the tibial tubercle and was thus used as an anatomic landmark to ensure the appropriate tensioning of the PT. One 5 mm metallic anchor (TWINFIX™ Ti, Smith & Nephew, Andover, USA), with two high resistance sutures, was placed 2.5 cm distal to the TT. Subsequently, a Krackow suture of the PT was performed, and the free ends of the suture were knotted. Then, one suture (ULTRATAPE™, Smith & Nephew, Andover, USA) was placed through the quadriceps tendon and in a figure-of-eight manner was secured beneath the TT to enforce the repair (Figures 3-4).

The wound was irrigated, and a multilayered closure was performed. The knee was immobilized with an extension brace. The patient was discharged two days later without complications.

**Figure 5:**
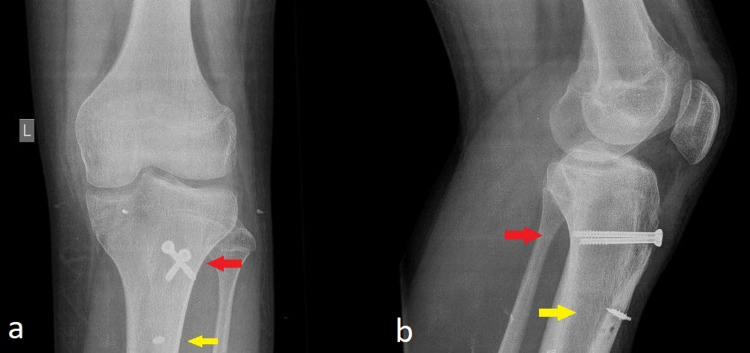
AP (a) and lateral (b) radiographs at five weeks postoperatively The two cannulated screws (red arrows) and the 5 mm anchor below the tibial tubercle (yellow arrows) are noted. AP - anteroposterior

On a follow-up, the patient visited our outpatient office two weeks postoperatively for wound inspection and stitch removal, and he started partial weight-bearing with crutches and a knee immobilization device. At four weeks, he was allowed to initiate passive flexion-extension of the knee to 30º with no resistance. The radiograph showed total consolidation of the fracture at five weeks postoperatively (Figure 5).

At six weeks, the knee immobilization device was removed. Subsequently, strengthening exercises of the quadriceps, such as passive flexion-extension movements to 90º of flexion and limited active flexion-extension movements of the limb (0º to 60º), were initiated; ambulation with full weight-bearing assisted by a crutch was allowed. At eight weeks, he was allowed to move freely with full weight-bearing. At 12 weeks, he could perform 130º of flexion and full extension. He was allowed to return to sports activities at five months postoperatively, and at the last follow-up (one year postoperatively), the patient reported being in excellent condition. 

## Discussion

Simultaneous TTF and PTR are rare, mostly attributed to excess forces during sports. The occurrence of such injuries has increased due to the growing participation in sports at younger ages. Very few cases have been reported in the literature [3]; hence suspicion of such complex injuries should be high. Therefore, we present a case of combined simultaneous TTF along with PTR and discuss the related literature.

TTF occurs due to failure through the secondary growth plate of the proximal tibia and is more commonly seen in boys aged 12-17 [1-3]. It is usually attributed to jumping actions that most commonly occur during sports participation, with basketball being the most common [3], like in our case. Forceful quadriceps contraction, particularly during jumping activities, imposes tremendous forces on the more distal part of the extensor mechanism. This eccentric contraction possibly results in the avulsion of the TT since the secondary growth plate is the most vulnerable site of the extensor apparatus [2]. However, the exact mechanism for the simultaneous patellar tendon rupture remains unclear [4-6]. The synchronous failure of TT and PT may imply perfectly balanced forces at both sites [7]. Moreover, if the TT bony fragment has been rotated 180°, like in our case, there is apparently increased resistance of the extensor mechanism that leads to the PTR, provided that the quadriceps contraction is maintained [8,9].

Diagnosis is based on both clinical and radiological findings. Loss of active knee extension and presence of patella alta in lateral knee X-ray should raise suspicion for PTR. Increased patella-to-tibia tubercle distance in a flexed position in lateral knee X-ray indicates combined TT and PT injury. Insall-Salvati and Caton-Deschamps indexes have also been used [3]. MRI aids in confirming the diagnosis [10]; however, it is not available in all institutions. The TTF is classified by Ogden et al. into types I, II, and III and subtypes A and B depending on the degree of comminution [1]. Frankl et al. added a subtype C with associated PTR; our patient had a type IIC injury [5].

The detection of PTR in patients with TTF is important as this directly impacts the management plan. More specifically, late, neglected, or, even worse, missed diagnosis leads to increased rates of reoperations to address the PTR. Panagopoulos et al. recently reported a case of missed PTR combined with TTF in a 15-year-old male after falling during cycling [11]. The TTF was successfully treated with open reduction and internal fixation (ORIF); however, the patient could not raise his leg straight after the operation. Postoperative radiologic workup revealed the characteristic patella alta, which confirmed the diagnosis of the PTR. Subsequently, the patient underwent a second procedure to address the neglected PTR.

Multiple treatments and fixation methods for the combination of TTF and PTR have been proposed and used [3]. However, we believe that a stable TT fixation along with repair and adequate reinsertion of the PT to its insertion site can have satisfactory short- and long-term results. While most researchers agree on open reduction and internal fixation of the TT fragment, reinsertion of the PT has been described using many techniques such as staples, trans-osseous suturing, figure-of-eight tension band wiring, and cerclage wire or suture anchor augmented with protective wire loop [10]. Nevertheless, the choice of treatment plan depends on the patient's age, the comminution of the TT fragment, and the surgeon's preference and experience.

Long-term complications, such as genu recurvatum and leg-length discrepancies due to growth plate injury, have also been reported [12]. It is, therefore, crucial to follow up with the patients until they reach skeletal maturity. 

## Conclusions

Simultaneous TTF and PTR is a rare and complex injury that requires prompt diagnosis and treatment. A high index of suspicion is needed with TTFs for simultaneous PT injury, especially with the absence of flexor knee mechanism and the presence of patella alta. Detection of PTR is crucial as it changes the treatment plan. 
